# Postoperative pneumothorax occurrence in patients with Marfan syndrome

**DOI:** 10.1007/s11748-025-02142-1

**Published:** 2025-04-10

**Authors:** Masahiro Yanagiya, Jun Nakajima, Chihiro Konoeda, Masaaki Sato

**Affiliations:** Department of Thoracic Surgery, The University of Tokyo Graduate School of Medicine, 7 - 3- 1 Hongo, Bunkyo-ku, Tokyo, 113 - 8655 Japan

**Keywords:** Pneumothorax, Marfan syndrome, Thoracic surgery, Chest deformity

## Abstract

**Objectives:**

Marfan syndrome is a congenital connective tissue disorder frequently complicated by pneumothorax. However, the long-term efficacy of surgical intervention in these cases remains underreported. This study aimed to assess the surgical outcomes of pneumothorax associated with Marfan syndrome.

**Methods:**

A retrospective review was conducted at a single center, including patients diagnosed with secondary pneumothorax associated with Marfan syndrome who underwent surgery between 2004 and 2019. Postoperative pneumothorax recurrence rates and potential predictors of postoperative recurrence were assessed. The incidence of ipsilateral pneumothorax after surgery was analyzed and compared with that before surgery.

**Results:**

Overall, 20 patients (60% male, 40% female) with a median age of 18.5 years (range 13–40 years) were included in the analysis. Seventeen patients underwent bullectomy, while the remainder received pulmorrhaphy. The cumulative incidence of postoperative pneumothorax at 5 and 10 years was 25% and 44%, respectively. Notably, patients with pectus excavatum had a 5-year cumulative recurrence rate of 25%, and those with a flat chest had a rate of 60%, compared with 9.1% for patients without chest deformities (P = 0.017). Chest deformity emerged as a significant predictor of postoperative pneumothorax recurrence (hazard ratio 7.63; 95% confidence interval 1.29–45.1; P = 0.025). The frequency of ipsilateral pneumothorax significantly decreased postoperatively, from 1.09 ± 0.66 episodes/year (mean ± SD) pre-surgery to 0.04 ± 0.10 episodes/year post-surgery (*P* < 0.001).

**Conclusions:**

Surgical intervention is an effective treatment for pneumothorax in patients with Marfan syndrome. Chest deformity may serve as a predictor of postoperative pneumothorax recurrence in this patient population.

## Introduction

Marfan syndrome is a congenital connective tissue disorder that affects multiple organ systems, including the cardiovascular, skeletal, and ophthalmologic systems [1]. Pneumothorax is a recognized pulmonary complication of Marfan syndrome, with an estimated incidence of 4–11% among affected individuals [2, 3]. Although several studies and case reports have indicated favorable outcomes with the surgical management of pneumothorax in patients with Marfan syndrome [4–6], the efficacy of surgery and long-term postoperative outcomes remain inadequately studied due to the rarity of the condition. This study aimed to assess the surgical outcomes of pneumothorax in patients with Marfan syndrome.

## Subjects and methods

### Patients and data acquisition

This retrospective study analyzed patients diagnosed with secondary pneumothorax associated with Marfan syndrome who required inpatient treatment between January 2004 and December 2019 at our institution. Patients who did not undergo surgical treatment for pneumothorax were excluded from the analysis.

Our clinical practice follows conventional treatment guidelines [7]. Briefly, surgery was indicated for patients who had recurrent pneumothorax, persistent air leakage, bilateral pneumothorax, or hemothorax [7]. If patients experienced the first episode of pneumothorax without persistent air leakage or hemothorax, surgery was offered for those who requested it.

In our institute, we perform bullectomy or pulmorrhaphy for spontaneous pneumothorax. Depending on the surgeon’s preference, a polyglycolic acid sheet is covered on the staple line or visceral pleura for reinforcement to reduce the postoperative recurrence rate.

The primary study endpoint was the cumulative incidence of postoperative pneumothorax recurrence, defined as any occurrence of ipsilateral or contralateral pneumothorax after surgery.

The secondary study endpoint was the frequency of postoperative ipsilateral pneumothorax compared with preoperative occurrence, as previously described [8]. To assess the efficacy of the surgical interventions, the number of ipsilateral pneumothorax episodes per patient was evaluated. The frequency of ipsilateral pneumothorax was calculated by dividing the number of ipsilateral pneumothorax episodes by the observation time (in years) [8]. The preoperative observation period was defined as the time from the first pneumothorax episode to the initial surgery date [8]. The postoperative observation period was defined as the time from the initial surgery date to the last follow-up [8]. In cases where patients underwent simultaneous bilateral surgery for synchronous bilateral pneumothorax, the frequency was calculated based on the side with the greater number of pneumothorax episodes for each patient.

The tertiary study endpoints were the details of postoperative recurrence of ipsilateral and contralateral pneumothorax. The cumulative incidences of postoperative ipsilateral and contralateral pneumothorax recurrence were calculated respectively. Treatments for those who experienced recurrence were also investigated.

**Fig. 1:**
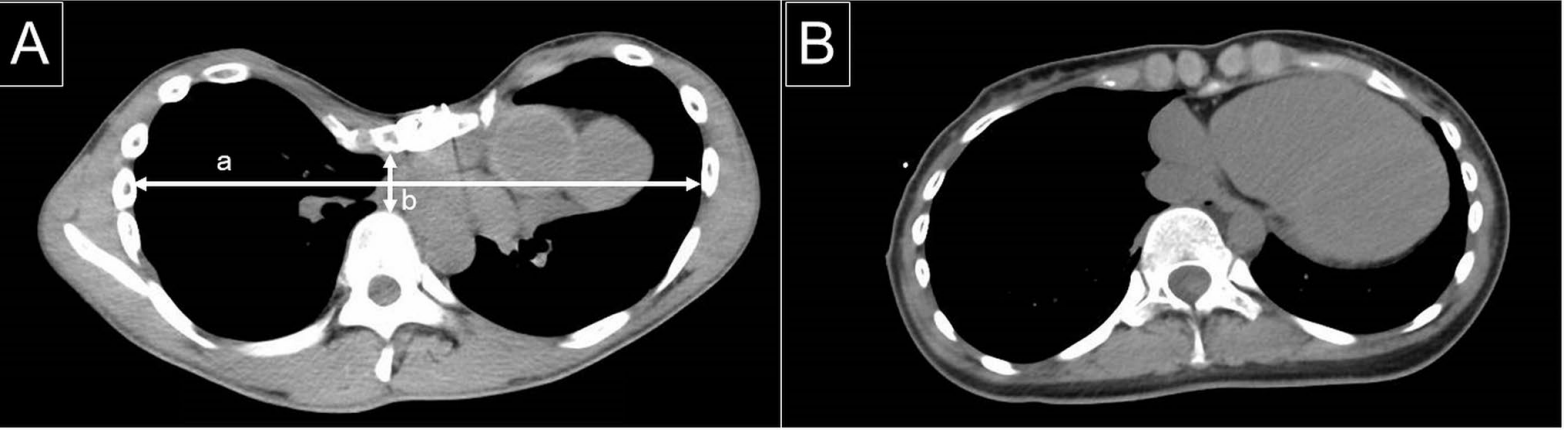
Chest deformity. **A** A typical image of pectus excavatum in an eligible patient. The Haller index was calculated as the ratio of the transverse diameter (**a**) to the anteroposterior diameter (**b**). **B** A typical image of a flat chest in an eligible patient

The following variables were analyzed as potential risk factors for pneumothorax recurrence: age at the time of initial surgery, sex, chest deformity, and use of a polyglycolic acid sheet. Chest deformity was defined as either pectus excavatum or flat chest. Pectus excavatum was identified based on a Haller index of ≥ 3.25 as previously reported (Fig. 1A) [9]. Flat chest was defined as a chest wall configuration with an anteroposterior diameter to transverse diameter ratio of < 1/3 at the level of the 8 th vertebra, as reported previously (Fig. 1B) [10]. The Haller index was initially calculated for patients suspected of having pectus excavatum. If the criteria for pectus excavatum were met, the patient was assigned to the pectus excavatum group. Subsequently, the anteroposterior diameter to transverse diameter ratio at the 8 th vertebra was measured in the remaining patients, and those meeting the criteria for flat chest were assigned to the flat chest group.

All data were obtained through a retrospective review of institutional medical records. The final date of follow-up for this study was December 31, 2023. Chest deformities were assessed using computed tomography imaging at the time of the initial surgery. Complications were defined according to the Clavien–Dindo classification, with grade III or higher considered significant [11].

### Statistical analysis

To assess the cumulative incidence of pneumothorax, deaths from any cause were treated as competing events. Comparisons of the cumulative incidence of postoperative pneumothorax across different groups were conducted using Gray’s test, with Bonferroni correction applied to adjust empirical *p *values for multiple comparisons. Competing-risk regression models, based on the Fine and Gray method, were utilized to identify predictive factors significantly affecting the risk of pneumothorax occurrence, incorporating competing events into the analysis. Hazard ratios (HRs) for recurrence, accompanied by 95% confidence intervals (CIs), were calculated. Univariate and multivariate regression models were applied to identify significant risk factors associated with pneumothorax occurrence.

The frequency of pneumothorax episodes per year, pre- and post-surgery, was analyzed using a paired *t* test [8]. Additionally, Poisson regression analysis was used to compare the frequency of pneumothorax occurrence before and after surgery, following the methodology outlined in the cited report [8].

All statistical analyses were performed using EZR (Saitama Medical Center, Jichi Medical University, Saitama, Japan, 2012), a graphical user interface for R (The R Foundation for Statistical Computing, Vienna, Austria, version 2.13.0) [12]. EZR is a modified version of R Commander (version 1.6–3) that incorporates additional biostatistical functions. Poisson regression analysis was conducted with JMP 12.0.1 (SAS Institute Inc., 2015). Statistical significance was set at *P* < 0.05.

### Ethical considerations

This study was approved by the Ethics Committee of the University of Tokyo Hospital (No. 2406–9). Given the retrospective nature of this chart review, the requirement for written informed consent was waived.

## Results

### Patient characteristics

Between January 1, 2004, and December 31, 2019, a total of 517 pneumothorax-related procedures were performed on 461 patients at our institution. After excluding 15 patients diagnosed with traumatic or iatrogenic pneumothorax, 446 patients were identified as having spontaneous pneumothorax. Among these, 22 patients were diagnosed with Marfan syndrome, and of these, 20 patients underwent surgical intervention for spontaneous pneumothorax at our hospital. The two patients who did not require surgery experienced a first episode of pneumothorax that resolved spontaneously with chest tube drainage. One patient had recurrence of ipsilateral pneumothorax 3 years after the first episode, but did not want to undergo surgery.

Twenty patients who underwent surgery were eligible for the current study. The demographic and clinical characteristics of the eligible patients are summarized in Table 1. The cohort comprised 12 men (60%) and 8 women (40%), with a median age of 18.5 years (range 13–40 years). All cases were assessed by cardiologists and diagnosed with Marfan syndrome prior to surgery. The prevalence of comorbidities is also presented in Table 1. Regarding chest deformities, pectus excavatum was observed in 4 patients, and 5 patients exhibited a flat chest.

**Table 1:** Baseline demographics of patients

Median age (years) [range]	18.5 [13–40]
Female sex, % (n)	40.0% (8)
Diagnosis	
Confirmed by genetic testing	11
Not examined with genetic testing	4
Unknown	5
Comorbidities, n	
Cardiovascular disease	20
Annuloaortic ectasia	13
History of aortic dissection	2
Mitral valve prolapse	4
Mitral regurgitation	1
Ophthalmologic disease	10
Lens dislocation	8
Flat cornea	2
Family history of Marfan syndrome, n	
Yes	10
No	6
Unknown	4
Chest deformity, n	
Pectus excavatum	4
Flat chest	5

Because all cases eligible for the current study had been seen by cardiologists, we summarized the details of their cardiovascular surgeries in Table 2. Twelve patients (60%) underwent cardiovascular surgery. Four patients (25%) had left thoracotomies because of cardiovascular surgery. Seven patients (35%) had a history of cardiovascular surgery prior to pneumothorax surgery (Table 2). Two patients (10%) had undergone cardiovascular surgery prior to their first episode of pneumothorax (Table 2), with both surgeries performed via median sternotomy. Only one patient, who later developed right-sided spontaneous pneumothorax, had previously undergone left thoracotomy due to cardiovascular surgery before the pneumothorax surgery.

**Table 2:** Details of cardiovascular surgery

History of cardiovascular surgery, n	
Yes	12
No	8
Approach, n	
Median sternotomy	12
Left thoracotomy	4
Timing of cardiovascular surgery, n	
Before pneumothorax surgery	7
At the same time of pneumothorax surgery	2
After pneumothorax surgery	10
Before first episode of pneumothorax	2

Overlap included. The “n” indicates the number of patients

In the present study, the preoperative observation period ranged from 1.0 to 189 months, with a median of 4.1 months (interquartile range [IQR] 1.0–47.8 months; mean 28.1 months). Postoperative follow-up ranged from 10.1 to 231 months, with a median of 97.2 months (IQR 81.3–156 months; mean 113 months).

### Indications for surgery

**Fig. 2:**
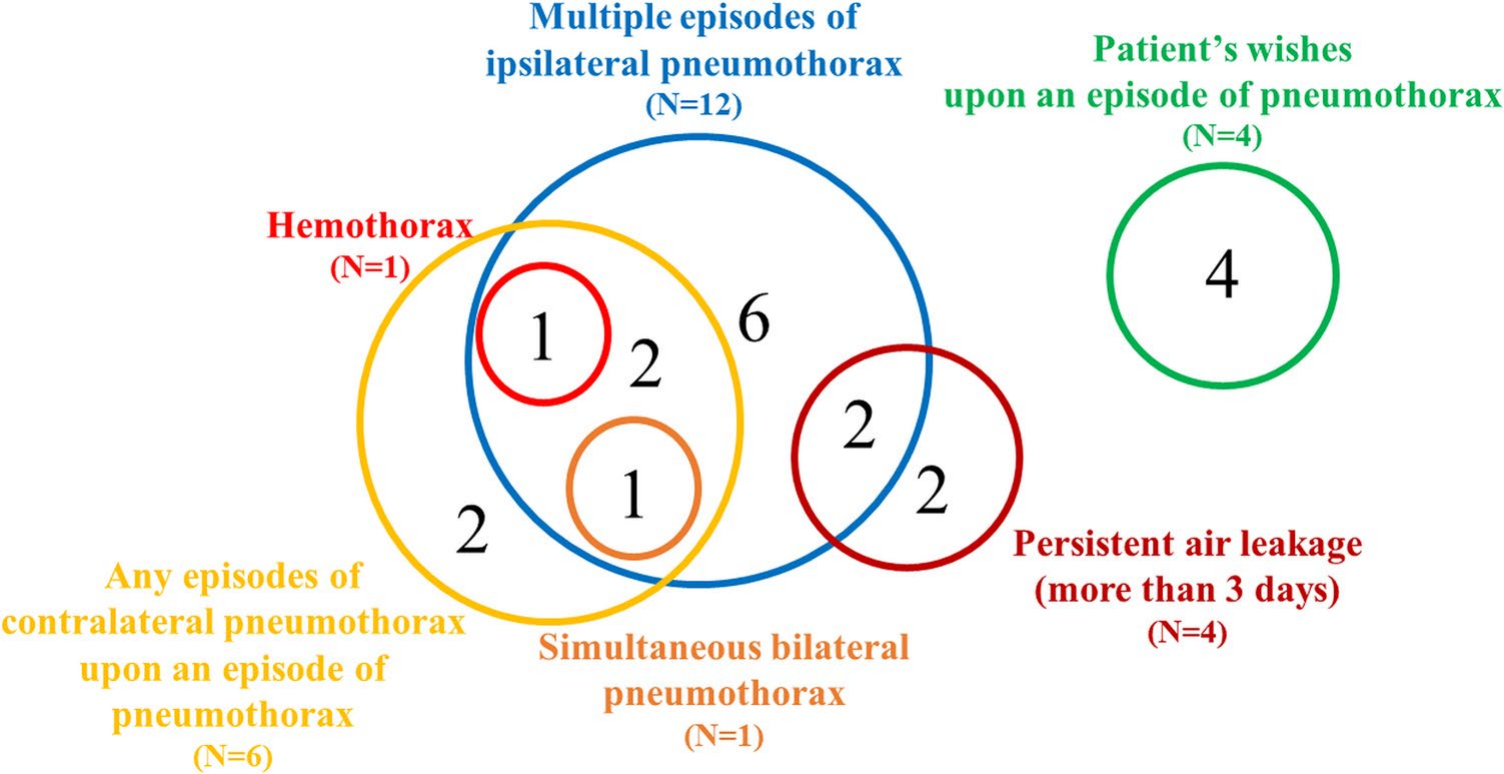
Surgical indications for pneumothorax in patients with Marfan syndrome. The numbers of patients with each indication are summarized in a Venn diagram

The indications for surgery are summarized in Fig. 2. Sixteen patients (80%) had at least one of the indications including multiple episodes of ipsilateral pneumothorax, any episodes of contralateral pneumothorax, persistent air leakage (more than 3 days), simultaneous bilateral pneumothorax and hemothorax, whereas the other patients (20%) underwent surgery at their own request. Elective surgery, defined as scheduled in outpatient services, was conducted in two patients (10%), whereas the other patients (90%) underwent semielective or emergency surgery. The number of episodes of pneumothorax before surgery is summarized in Table 3. Twelve patients (60%) had at least two episodes of pneumothorax before surgery.

**Table 3:** Summary of the number of episodes of pneumothorax before surgery

Number of episodes before surgery, n	Number of patients, n (%)
0	0 (0)
1	8 (40)
2	9 (45)
3	2 (10)
4	1 (5)

The number of episodes of pneumothorax before surgery includes the episode which finally indicated surgery

### Characteristics of pneumothorax and surgery

The characteristics of pneumothorax and the surgical procedures performed are summarized in Table 4. Bullae were localized to the apex in 15 patients (75%), multiple bullae were observed in seven patients (35%), and all bullae were treated during surgery. Video-assisted thoracic surgery was employed in 90% of cases. Two patients (10%), who underwent open thoracotomy had simultaneous cardiac or aortic surgery in addition to pneumothorax surgery. Seventeen patients (85%) underwent bullectomy, and the remaining patients underwent pulmorrhaphy. The application of polyglycolic acid sheet was performed in 17 patients (85%) to reinforce the staple line or visceral pleura. Two patients (10%) experienced complications necessitating additional invasive therapeutic interventions (Table 4). One patient required thoracentesis for pleural effusion, whereas the other underwent surgery for postoperative air leakage.

**Table 4:** Summary of pneumothorax and surgery

Pneumothorax, n	
Right-sided	10
Left-sided	9
Bilateral	1
Location of pulmonary bullae	
Apex	15
Apex, interlobar fissure of upper lobe	1
Superior segment of lower lobe	1
Apex and superior segment of lower lobe	1
Interlobar fissure of upper lobe, superior segment of lower lobe, and lingular segment	1
Azygoesophageal recess	1
Surgical approach, n	
VATS	18
Open thoracotomy	2
Surgical procedure, n	
Bullectomy	17
Pulmorrhaphy	3
Use of polyglycolic acid sheet, n	
Yes	17
No	3
Complication, n	
Pleural effusion	1
Persistent air leak	1

*VATS* Video-assisted thoracic surgery

### Cumulative incidence of postoperative pneumothorax

**Fig. 3:**
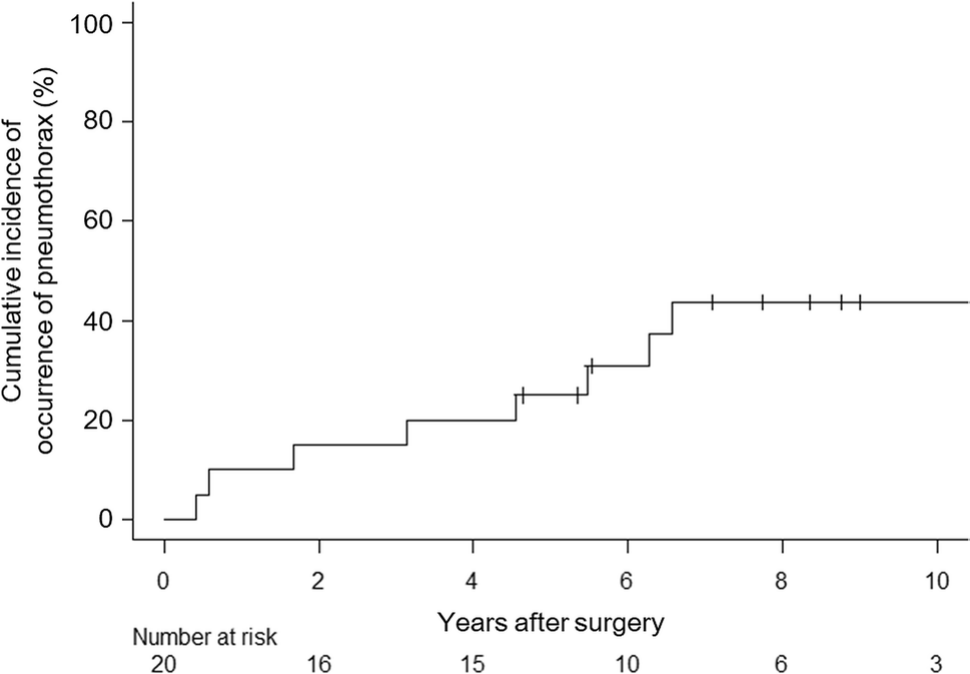
Cumulative incidence of the overall pneumothorax occurrence after surgery

**Fig. 4:**
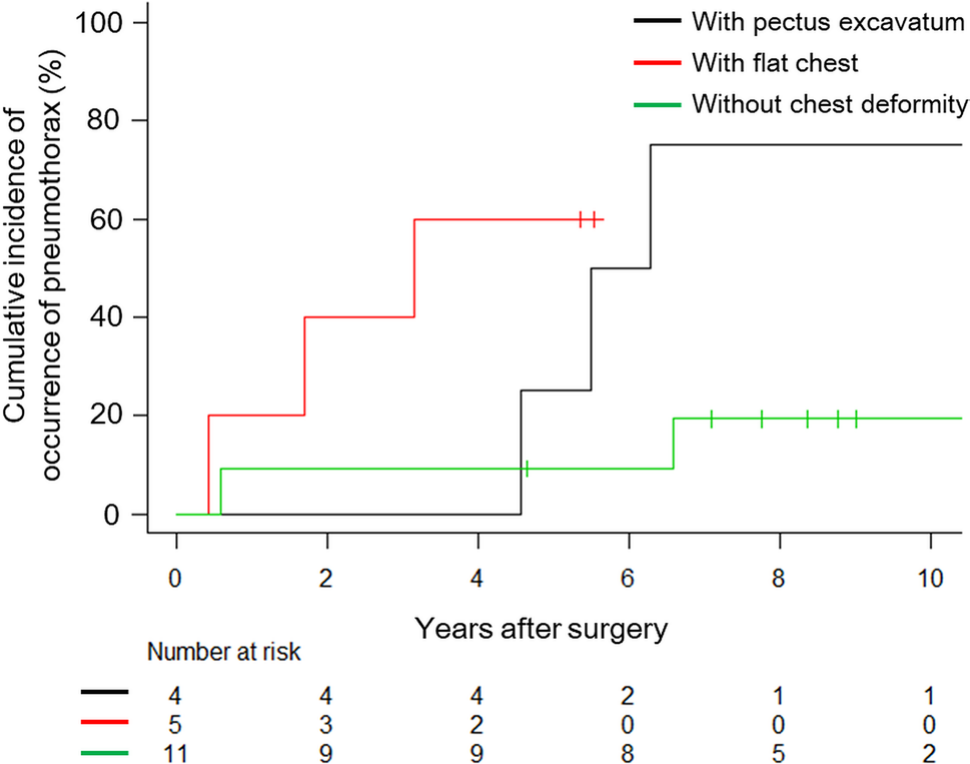
Cumulative incidence of the overall pneumothorax occurrence after surgery based on the presence of chest deformity. Gray’s test indicated a significant difference in the cumulative incidence of postoperative pneumothorax occurrence (*P* = 0.017). Patients with pectus excavatum had a significantly higher cumulative incidence of postoperative pneumothorax than those without chest deformity (*P* = 0.034), with Bonferroni correction applied for multiple comparisons

The cumulative incidence of overall pneumothorax at 5 and 10 years post-surgery was 25% and 44%, respectively, for the entire cohort (Fig. 3). When stratified by chest morphology, the 5-year cumulative incidence of postoperative pneumothorax was 25% for patients with pectus excavatum, 60% for those with a flat chest, and 9.1% for patients without any chest deformity (*P* = 0.017) (Fig. 4). After applying Bonferroni correction for multiple comparisons, the postoperative pneumothorax incidence remained significantly higher in patients with pectus excavatum compared with those without chest deformity (*P* = 0.034) (Fig. 4).

### Impact of chest deformity on postoperative pneumothorax

Univariate analysis identified chest deformity as a significant factor associated with postoperative pneumothorax (Table 5). Further, multivariate analysis, including the use of a polyglycolic acid sheet, confirmed that chest deformity remained a significant predictor for postoperative pneumothorax (HR 7.63; 95% CI 1.29–45.1; P = 0.025) (Table 5).

**Table 5:** Risk factors for the postoperative occurrence of pneumothorax

	Univariate analysis			Multivariate analysis		
Factors	Hazard ratio	95% CI	*P* value	Hazard ratio	95% CI	*P* value
Age	0.894	0.787–1.015	0.083	0.908	0.823–1.002	0.055
Sex, male vs. female	2.294	0.513–10.26	0.280	1.877	0.711–4.955	0.200
Chest deformity, yes vs. no	8.314	1.737–39.79	0.008	7.630	1.290–45.13	0.025
PGA sheet, yes vs. no	0.790	0.199–3.131	0.740	1.625	0.528–5.002	0.400

*CI* confidence interval, *HR* hazard ratio, *PGA* polyglycolic acid

### Frequency of ipsilateral pneumothorax after surgery

**Fig. 5:**
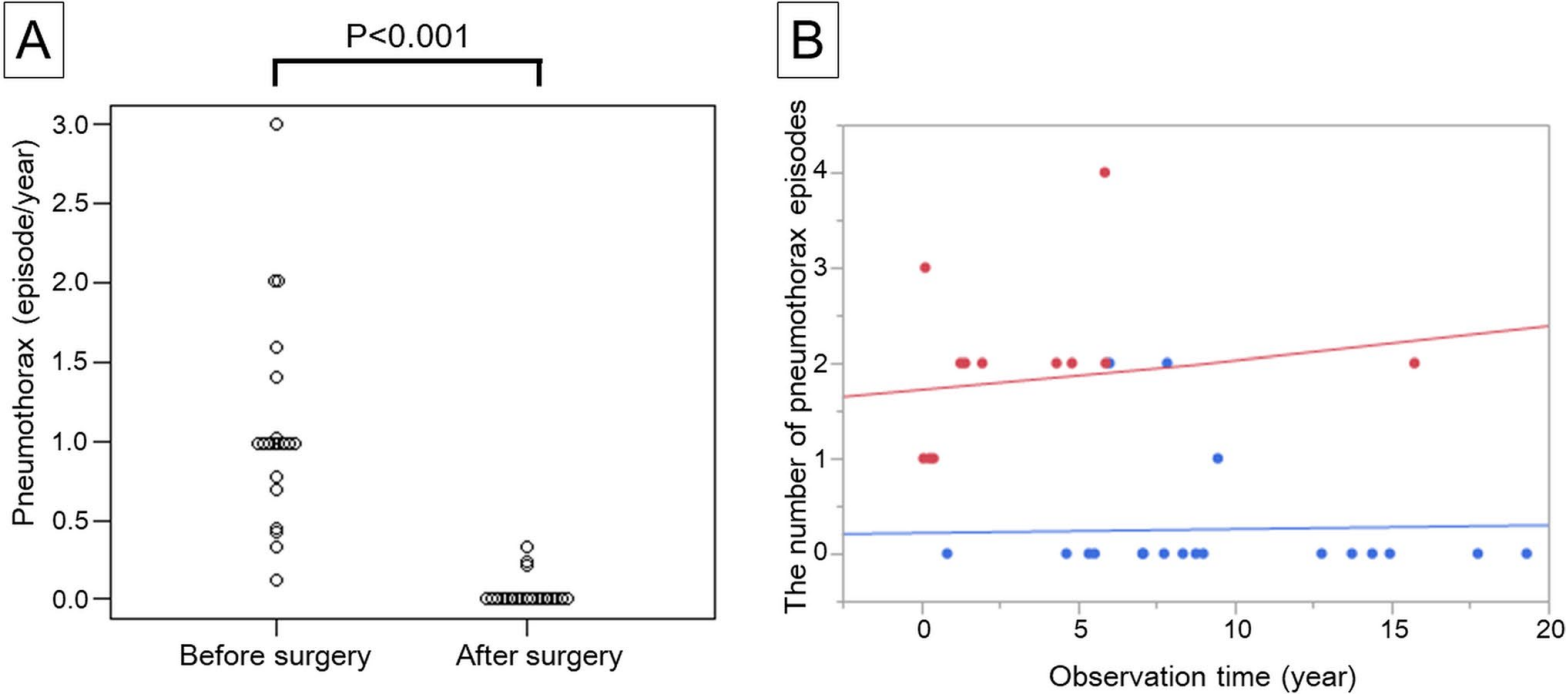
Comparison of the frequency of ipsilateral pneumothorax episodes before and after surgery. **A** The frequency of ipsilateral pneumothorax episodes was calculated as episodes per year by dividing the number of episodes by the observation time (years from the first pneumothorax episode to surgery or from the initial surgery date to the last follow-up). A significant reduction in the frequency of ipsilateral pneumothorax (episodes/year) was observed after surgery. **B** A Poisson regression model was used to compare the number of ipsilateral pneumothorax episodes before surgery (red dot) and after surgery (blue dot). The estimated regression equation for the number of episodes before surgery was e^0.539+0.017*years^ (red line) and after surgery was e^−1.544+0.017*years^ (blue line)

**Fig. 6:**
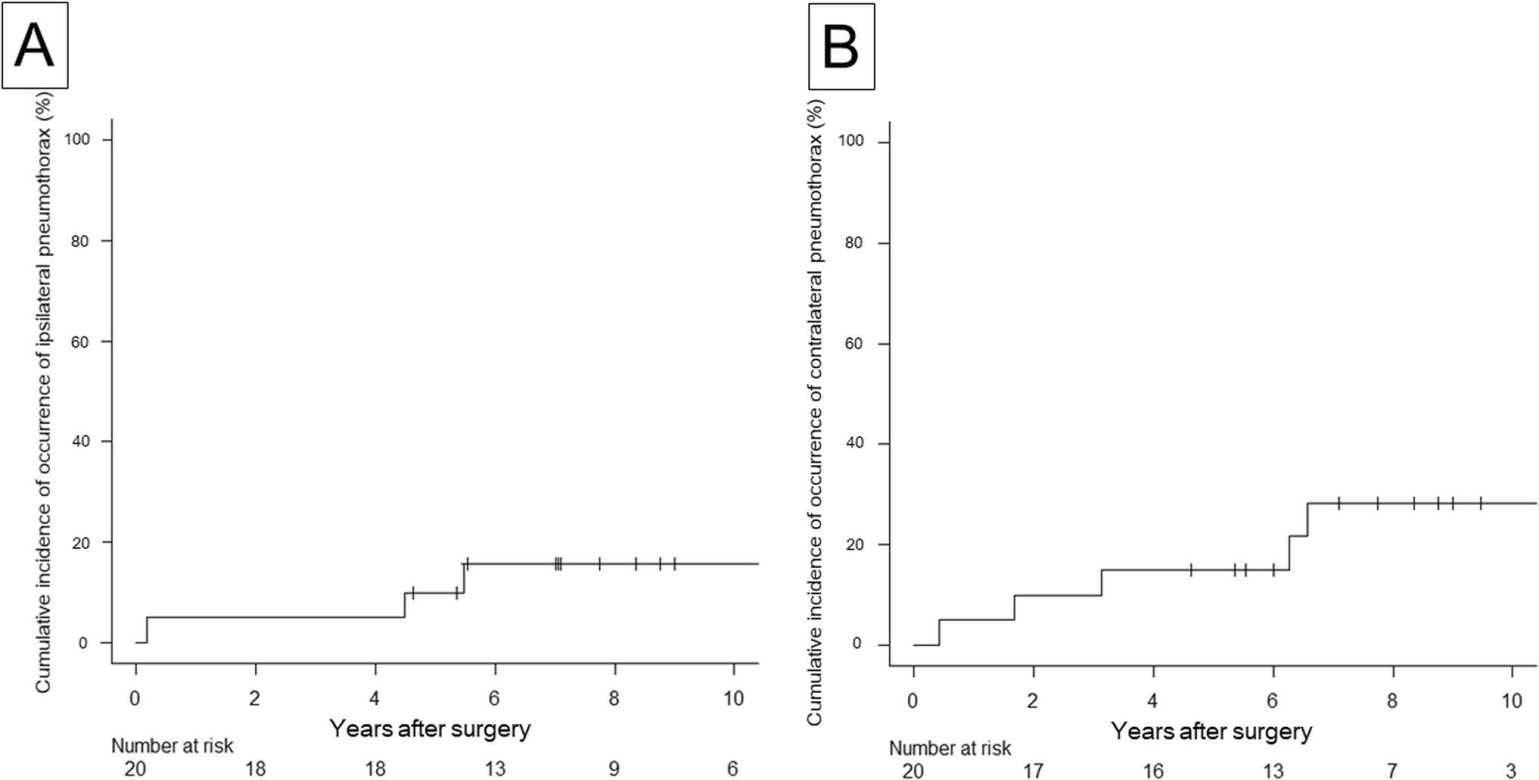
Cumulative incidence of ipsilateral and contralateral pneumothorax occurrence after surgery. **A** Cumulative incidence of ipsilateral pneumothorax occurrence after surgery. **B** Cumulative incidence of contralateral pneumothorax occurrence after surgery

The incidence of ipsilateral pneumothorax following surgical intervention was markedly reduced, with a post-surgery frequency of 0.04 ± 0.10 episodes per year (mean ± SD) compared to a pre-surgery frequency of 1.09 ± 0.66 episodes per year (*P* < 0.001) (Fig. 5A). Poisson regression analysis indicated a significant reduction in the frequency of ipsilateral pneumothorax postoperatively, with an estimated effect size of – 2.082 (95% CI – 3.265 to – 1.049; *P* < 0.001) (Fig. 5B). The cumulative incidence of ipsilateral pneumothorax was 10% at 5 years and 16% at 10 years (Fig. 6A). The postoperative recurrence of ipsilateral pneumothorax was documented in three patients, all of whom experienced two episodes of pneumothorax after surgery. Surgery was conducted for all three patients who experienced a recurrence of ipsilateral pneumothorax.

We conducted a subgroup analysis to evaluate whether surgery was effective to reduce episodes of recurrent pneumothorax for patients who had experienced multiple episodes of pneumothorax before surgery. As shown in Fig. 2, 12 patients had experienced multiple episodes of ipsilateral pneumothorax before surgery. Among them, patients who underwent surgery for persistent air leakage or hemothorax were excluded. Nine patients were eligible in this subgroup analysis. Regarding this subgroup, the incidence of ipsilateral pneumothorax following surgical intervention was also reduced, with a post-surgery frequency of 0.02 ± 0.06 episodes per year (mean ± SD) compared to a pre-surgery frequency of 1.25 ± 0.86 episodes per year (P = 0.002).

In addition, we performed another subgroup analysis to evaluate whether surgery was effective for patients with chest deformity. Regarding 9 patients with chest deformity, the incidence of ipsilateral pneumothorax following surgical intervention was reduced, with a post-surgery frequency of 0.05 ± 0.10 episodes per year (mean ± SD) compared to a pre-surgery frequency of 1.45 ± 0.72 episodes per year (P < 0.001). The 5-year cumulative incidence of ipsilateral pneumothorax after surgery for patients with chest deformity was not significantly higher than those without chest deformity (11.1% vs. 9.1%, P = 0.46).

### Postoperative occurrence of contralateral pneumothorax

The cumulative incidence for contralateral pneumothorax was 15% at 5 years and 28% at 10 years (Fig. 6B), which was not significantly higher than that for ipsilateral pneumothorax (P = 0.25). Contralateral pneumothorax was documented in six patients. Of these, five patients underwent surgical intervention, while one case resolved spontaneously under outpatient observation. Recurrent contralateral pneumothorax necessitated an additional surgical procedure in one patient. No further recurrences were observed in other patients following the resolution of their initial contralateral pneumothorax episode.

**Fig. 7:**
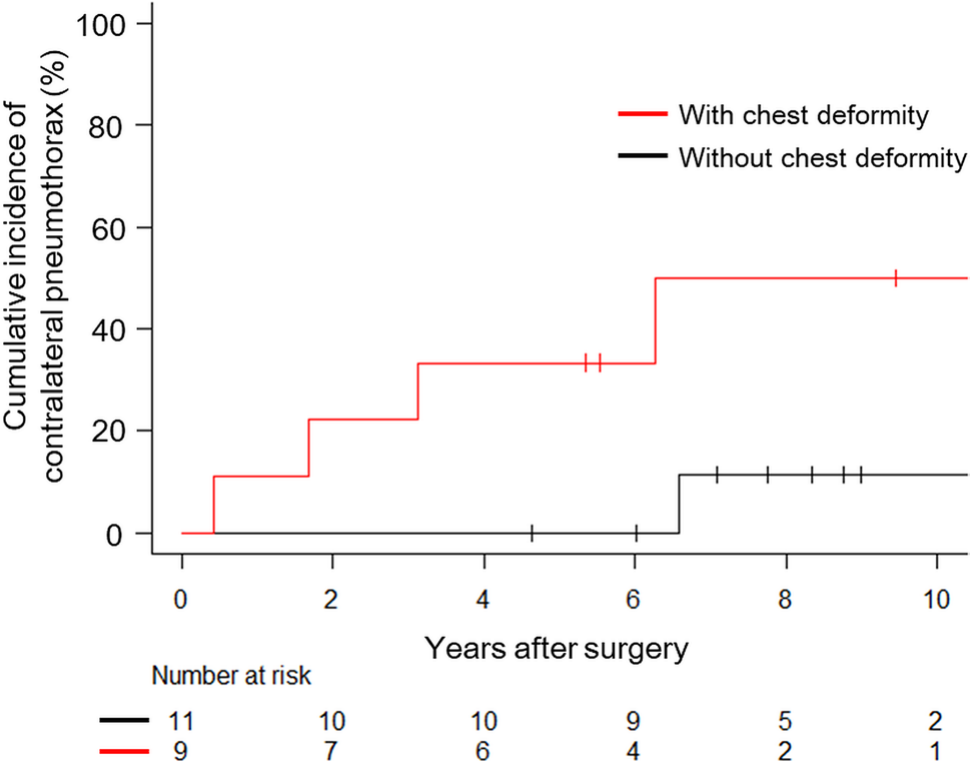
Cumulative incidence of contralateral pneumothorax occurrence after surgery for patients with and without chest deformity. The cumulative incidence of contralateral pneumothorax for patients with chest deformity at 5 and 10 years post-surgery was 33% and 50%, respectively, while that for those without chest deformity at 5 and 10 years was 0% and 11%. There was a significant difference between the cumulative incidence of contralateral pneumothorax for patients with and without chest deformity (P = 0.02)

We evaluated cumulative incidences for contralateral pneumothorax with and without chest deformity. The cumulative incidence of contralateral pneumothorax for patients with chest deformity at 5 and 10 years post-surgery was 33% and 50%, respectively, which was significantly higher than those without chest deformity (P = 0.02) (Fig. 7).

## Discussion

Our findings suggest that surgical intervention is an effective treatment for pneumothorax secondary to Marfan syndrome. In addition, surgical intervention would be also effective to reduce recurrent pneumothorax for patients who experienced multiple episodes of ipsilateral pneumothorax. As illustrated in Fig. 5, the incidence of ipsilateral pneumothorax significantly decreased following surgical intervention. The 5-year cumulative incidence of postoperative ipsilateral pneumothorax was 10% (Fig. 6A), which was not significantly lower than that of contralateral pneumothorax. Considering the incidence of contralateral pneumothorax, surgical intervention would not be so effective to the point where surgery significantly reduces postoperative ipsilateral recurrence much more than contralateral recurrence. However, previous studies have reported recurrence rates of spontaneous pneumothorax ranging from 6 to 20% [13–15]. When compared with outcomes of primary spontaneous pneumothorax surgery, these results suggest that our outcomes are acceptable for preventing ipsilateral pneumothorax recurrence.

Although the 5-year cumulative incidence of contralateral pneumothorax was 15% (Fig. 6B), which is comparable with prior reports on primary spontaneous pneumothorax [16, 17], patients with Marfan syndrome appear to experience contralateral pneumothorax comparable to ipsilateral recurrence post-surgery, as shown in Fig. 6. This finding is corroborated by previous reports [6, 18]. One study reported a high incidence of bilateral pneumothoraces [6], whereas another observed that approximately 55% of young Marfan syndrome patients developed contralateral pneumothorax following initial treatment [18]. Taking into account ipsilateral recurrence and contralateral occurrence of pneumothorax, the 10-year cumulative incidence of postoperative pneumothorax was found to be 44% (Fig. 3), underscoring the importance of addressing this complication. The elevated recurrence and bilateral pneumothorax rates observed in patients with Marfan syndrome are likely attributable to underlying generalized connective tissue defects and degenerated elastic fibers [2, 6].

In this study, five of the six patients with contralateral pneumothorax underwent surgical intervention. Notably, with the exception of one case, all patients remained pneumothorax-free following resolution of the initial episode. This suggests that surgery may also be effective for contralateral pneumothorax.

Additionally, we identified chest deformity as a potentially significant predictor of postoperative pneumothorax occurrence in patients with Marfan syndrome. Prior research has shown that a considerable proportion of patients with pectus excavatum develop bulla formation and have a higher risk for spontaneous pneumothorax [19]. Another study indicated that a flat chest is frequently associated with primary spontaneous pneumothorax [20]. Chest deformities, such as pectus excavatum and flat chest, can result in heterogeneous alveolar pressure, potentially leading to pleural buckling and subsequent pneumothorax [19, 20]. As reported previously, an approximately 20-fold increase in pleural stress was observed in the apex of those with a flattened chest compared with those without a flattened chest [21]. In addition, lungs from those with Marfan syndrome had a pattern of distal acinar emphysema also known as paraseptal emphysema [22]. Heterogeneous alveolar pressure and extremely high pleural stress seen in chest deformities might easily damage the pleural surface of distal acinar emphysema and contribute to the high postoperative pneumothorax recurrence rate.

Although chest deformity was a significant factor for postoperative occurrence of pneumothorax, surgery would be effective in reducing ipsilateral occurrence of pneumothorax because incidence of ipsilateral pneumothorax following surgical intervention was significantly reduced from a pre-surgery frequency of 1.45 ± 0.72 episodes per year to 0.05 ± 0.10 episodes per year (mean ± SD) (P < 0.001). However, as shown in Fig. 7, chest deformity would be likely to cause contralateral pneumothorax more frequently. Considering these results, we should pay more attention to contralateral occurrence of pneumothorax for patients with chest deformity. Although we did not perform simultaneous bilateral thoracic surgery in order to prevent contralateral recurrence except for simultaneous bilateral pneumothorax, simultaneous bilateral thoracic surgery might be taken into consideration for patients with chest deformity [23]. However, simultaneous bilateral thoracic surgery would be unfeasible because patients with Marfan syndrome would have cardiovascular risks due to their nature.

Based on our results, we propose that surgical strategy for pneumthorax with Marfan syndrome should be similar to that for primary spontaneous pneumothorax [7] because surgery significantly reduced ipsilateral recurrent pneumothorax even for patients with multiple episodes of pneumothorax and with chest deformity.

Although surgical strategy for pneumothorax with Marfan syndrome should be similar to that for primary spontaneous pneumothorax, we believe that pneumothorax with Marfan syndrome is classified as secondary spontaneous pneumothorax. Primary spontaneous pneumothorax is defined as having no evidence of underlying lung disease whereas secondary pneumothorax defined as having underlying lung diseases [7]. As reported previously, lungs of patients with Marfan syndrome have a pattern of paraseptal emphysema [22]. Considering this issue, pneumothorax with Marfan syndrome is secondary one.

Bullae were predominantly located at the apex of the lungs (Table 4), consistent with prior reports [6]. There appeared to be no difference in the location characteristics of bullae between patients with and without Marfan syndrome [6].

Cardiovascular surgery did not seem to influence the occurrence of pneumothorax in this study. Only one patient, who underwent left thoracotomy for cardiovascular surgery, subsequently developed a right-sided spontaneous pneumothorax. Additionally, only two patients underwent cardiovascular surgery via median sternotomy prior to their first spontaneous pneumothorax episode.

This study had several limitations. First, it was a single-center, retrospective analysis, and therefore, prospective studies are needed for more robust data collection. Given the rarity of Marfan syndrome [24], assembling a large patient cohort is challenging, making multicenter studies crucial for future research. Second, all patients in our study had cardiovascular diseases that were treated or managed by cardiologists, which could introduce potential selection bias. Finally, the frequency of pneumothorax might have been biased by observation periods and the number of episodes.

## Conclusion

Surgical intervention appears to be effective in reducing the ipsilateral recurrence of pneumothorax in patients with Marfan syndrome. Surgical strategy for pneumthorax with Marfan syndrome should be similar to that for primary spontaneous pneumothorax. However, the incidence of postoperative pneumothorax remains high when considering contralateral pneumothorax occurrences. Notably, chest deformity may serve as a predictive factor for postoperative pneumothorax in this patient population.

## Data Availability

The datasets used and analyzed in the current study are available from the corresponding author upon reasonable request.
